# Radiation recall dermatitis induced by ibuprofen^⋆^

**DOI:** 10.1016/j.abd.2021.08.017

**Published:** 2023-03-24

**Authors:** Li-wen Zhang, Juan Wu, Lu Zheng, Tao Chen

**Affiliations:** a Department of Dermatovenereology, Chengdu Second People’s Hospital, Chengdu, Sichuan, China; b Sexually Transmitted Disease Institute, Shanghai Skin Disease Hospital, School of Medicine, Tongji University, Shanghai, China

Dear Editor,

A 31-year-old female presented with a 1-week history of burning, painful and pruritic skin eruptions affecting the left chest and axillary region. She had previously undergone a modified radical mastectomy and lymph node dissection for ductal breast carcinoma 1 year ago. Then, the patient has prescribed 24 weeks of chemotherapy with doxorubicin, cyclophosphamide, and paclitaxel. Two months ago, she was treated with 3-dimensional conformal radiation therapy (50 Gy: 25 fractions for five weeks). Mild erythema and scales with pruritus were observed at the end of radiotherapy and subsided with 0.05% desonide cream within several days. Six days before the eruptions appeared, she had taken ibuprofen orally for arthralgia. The inflammatory eruptions involved the areas previously treated with radiation and initially manifested well-demarcated rectangular erythema, edema, and tiny papules, followed by blisters and erosions (Fig. 1). The dermoscopy showed multiple brown circles along with the hair follicles as well as scales (Fig. 2). The histopathology revealed intraepidermal vesiculation, papillary dermal edema, and perivascular inflammatory infiltrate in the upper dermis (Fig. 3).

**Figure 1:**
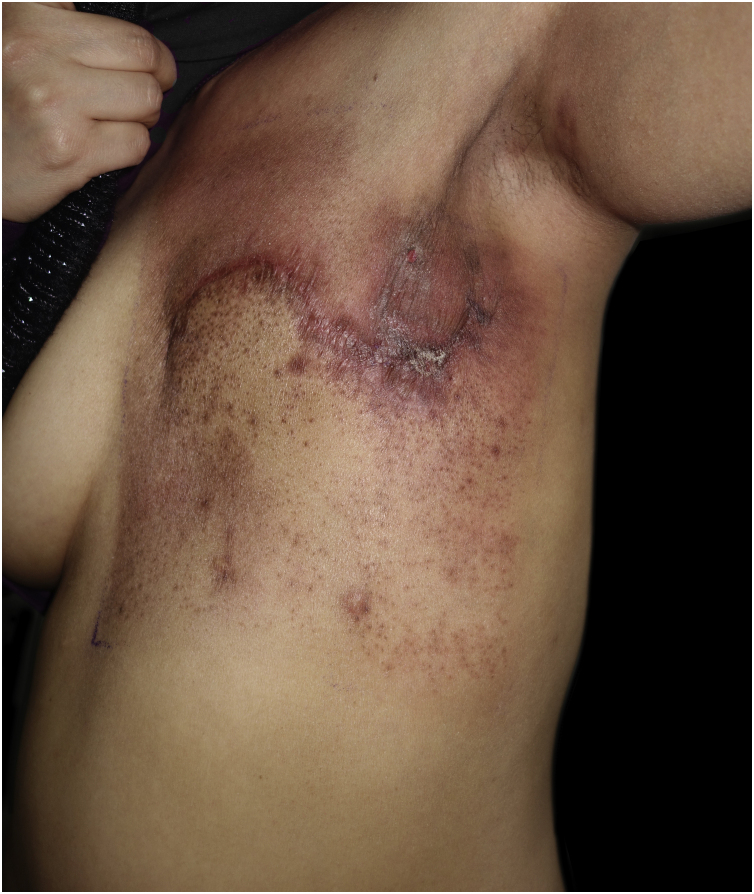
Well-demarcated rectangular erythema with edema, tiny papules, and erosions affecting the left chest and axillary region.

**Figure 2:**
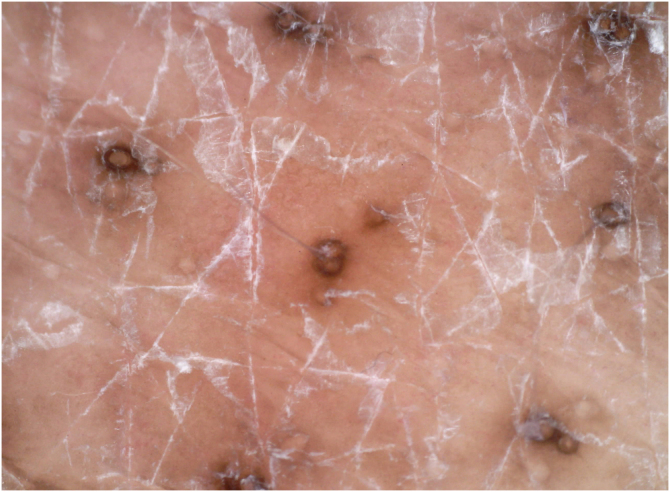
The dermoscopy showed multiple brown circles along with the hair follicles as well as white scales.

**Figure 3:**
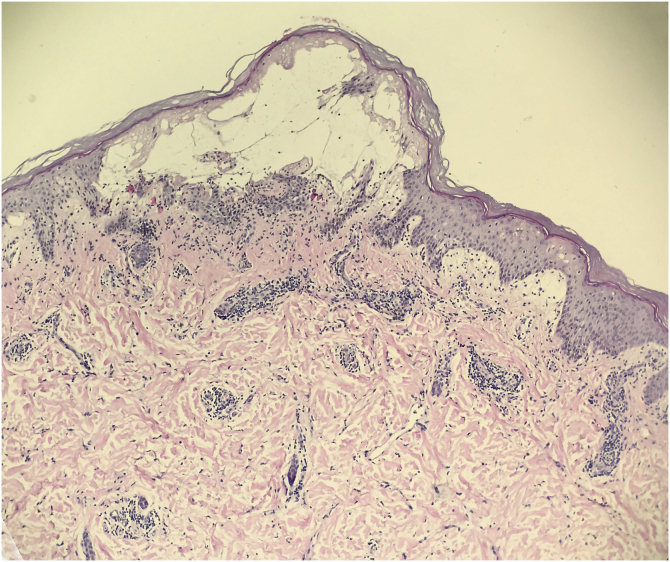
The histopathology revealed intraepidermal vesiculation, papillary dermal edema, and perivascular inflammatory infiltrate in the upper dermis (Hematoxylin & eosin, ×100).

A diagnosis of radiation recall dermatitis (RRD) induced by ibuprofen was made. The patient was relieved after 1 week of treatment with oral loratadine and topical 0.05% halometasone cream.

RRD is an uncommon acute dermatitis at regions of the previous radiotherapy in response to drug administration. The most commonly implicated medications are anticancer drugs, in particular cytotoxics, but other drugs can also induce RRD, including antibiotics, antituberculosis drugs, nimesulide, phentermine, and simvastatin.^1^ Chu et al. reported only one case of RRD following topical agents.^2^ RRD can occur at any time between days to years after radiation exposure. The time to develop the reaction may be longer for oral than intravenously administered drugs.^3^ RRD is drug-specific for any individual; it is impossible to predict which individuals will react to which drugs, and rechallenge does not uniformly cause a reaction.^1^ Several possible explanations for the pathogenesis of RRD have been proposed including memory reaction of surviving epithelial stem cells, radiation-induced mutation, post-radiation vascular damage, and hypersensitivity reaction.^3^

Most of the time, RRD presents as erythema, papules, edema, vesicles, desquamation, or even ulceration.^4^ The area affected by RRD corresponds to the site previously irradiated, although may occasionally become generalized. The histological appearance is nonspecific and sometimes overlaps with acute radiation dermatitis. It is important to note however, radiation recall reactions may involve deep tissues and organs.^1^

To our knowledge, there have been no reports of RRD induced by ibuprofen. This is the second case of RRD following a non-steroidal anti-inflammatory drug, and the first was induced by nimesulide.^5^ The dermoscopic manifestation shows a brown circle around the hair follicle that is similar to the dermoscopic appearance of lichen pilaris.

## Financial support

None declared.

## Authors’ contributions

Li-wen Zhang and Juan Wu contributed equally to this work.

Li-wen Zhang: Study conception and planning; preparation and writing of the manuscript.

Juan Wu: Literature review.

Lu Zheng: Acquisition of data.

Tao Chen: Approval of the final version of the manuscript.

## Conflicts of interest

None declared.
